# Supplementary material for: 3 minutes to precisely measure morphogen concentration
Source: PLoS Genet. 2018 Oct 26;14(10):e1007676. doi: 10.1371/journal.pgen.1007676 (PMC6221364; doi:10.1371/journal.pgen.1007676)
Supplement: S1 Text — (PDF) [file pgen.1007676.s001.pdf]

SI Text. Supplementary Molecular Biology

BAC recombineering: the *24xMS2* cassette was first cloned into a pL-452-N-eGFP plasmid (Addgene #19173) in 3' of a lox-Kan-lox selection cassette. Second, ~100 bp primers homologous to flanking regions of either the 5'-UTR or 3'-UTR regions of *hb* were generated to create recombination sites (respectively called 5'HR and 3'HR) and used to amplify 5'Fw-*24xMS2*-lox-KAN-lox-3'Rv PCR products. These PCR products were gel purified and electroporated into SW102 *E. coli* strain containing the *hb-18kb-BAC* (which contains a Chloramphenicol selection cassette) for recombination. The recombinant colonies were selected on Kanamycin (Kan) and Chloramphenicol (Cm) plates and the recombinant BAC was purified and electroporated into SW106 *E. coli* strains that carry an L-arabinose-inductible Cre gene for Kan selection cassette removal. The Kan^S Cm^R recombinant colonies were picked. The integrity of the MS2 loops sequence and the junctions of the insert at the recombination sites were verified by PCR, digestion and sequencing. The three BACs (*hb-18kb-BAC*, *5'MS2-18kb-BAC and 3'MS2-18kb-BAC*) were inserted by BestGene at the VK18 recombination site on the chromosome 2R (# 9736, Bloomington) [1].

HR Primers used for recombination within the 5'UTR or 3'UTR of *hb*.

5'UTR of *hb*	Fw	AAATGAAAAACAAGCGGAAAAAAAGAGGAAAAAACTCGACG CAGGCGCAGTGCATGAATGAATAAATGAATATGCCCACTAAC CCCACTCTCTCTtctcggcatggacgagctgtacaag
	Rv	TGTACCAGGCGTTGTGCTGCTCGTAGTTGGTCGTGGCTGTCGT CTCCCAGTTCTGCATCTTGGCGGCTCTAGACGGCTGTAATGGA TAAGAAAACgcggccgctctagaactagtggatc
3'UTR	Fw	TGTGCGGCGAGAAGTGCGACGGACCCGTCGGCCTCTTCGTTC ACATGGCCAGGAATGCTCACTCCTAAGTTCCCCATCACCATCA CCTTGTTATTtctcggcatggacgagctgtacaag
	Rv	GTACAATTTTCGACAACAAAATAATGTTTGGAAAACTTATGCT ACGAATATATACAATTCTGGACAACGATTATATGATAATAGT GATAAATAATgcggccgctctagaactagtggatc

Upper cases are homologous to *hb* sequence and lower cases are homologous to pL-452-N-eGFP plasmid

Sequence of the primers used to remove the Zelda binding site from the iRFP conding sequence: forward primer:AAAAAGATCTatggcgcgtaaggtcgatctcacctcctgcgatcgcgagccgatccac

atccccggcagcattcagccgtgcggctgtctcctagcctgcgacg, with the mutated Zelda binding site underlined; reverse primer: AAAAAGGATCCttagcgttggtggtgggcggcggtgaagtgc).

Sequence of the ΔZelda MS2 cassette:

5’GATCCTACGGTACTTATTGCCAAGAAAGCACGAGCATCAGCCGTGCCTCAATGTCGAATCT
GCAAACGACGACGATCACGCGTCGCTCCAGTATTCCAGGGTTCATCAGATCCTACGGTACTT
ATTGCCAAGAAAGCACGAGCATCAGCCGTGCCTCAATGTCGAATCTGCAAACGACGACGAT
CACGCGTCGCTCCAGTATTCCAGGGTTCATCAGATCCTACGGTACTTATTGCCAAGAAAGCA
CGAGCATCAGCCGTGCCTCAATGTCGAATCTGCAAACGACGACGATCACGCGTCGCTCCAGT
ATTCCAGGGTTCATCAGATCCTACGGTACTTATTGCCAAGAAAGCACGAGCATCAGCCGTGC
CTCAATGTCGAATCTGCAAACGACGACGATCACGCGTCGCTCCAGTATTCCAGGGTTCATCA
GATCCTACGGTACTTATTGCCAAGAAAGCACGAGCATCAGCCGTGCCTCAATGTCGAATCTG
CAAACGACGACGATCACGCGTCGCTCCAGTATTCCAGGGTTCATCAGATCCTACGGTACTTA
TTGCCAAGAAAGCACGAGCATCAGCCGTGCCTCAATGTCGAATCTGCAAACGACGACGATC
ACGCGTCGCTCCAGTATTCCAGGGTTCATCAGATCCTACGGTACTTATTGCCAAGAAAGCAC
GAGCATCAGCCGTGCCTCAATGTCGAATCTGCAAACGACGACGATCACGCGTCGCTCCAGTA
TTCCAGGGTTCATCAGATCCTACGGTACTTATTGCCAAGAAAGCACGAGCATCAGCCGTGCC
TCAATGTCGAATCTGCAAACGACGACGATCACGCGTCGCTCCAGTATTCCAGGGTTCATCAG
ATCCTACGGTACTTATTGCCAAGAAAGCACGAGCATCAGCCGTGCCTCAATGTCGAATCTGC
AAACGACGACGATCACGCGTCGCTCCAGTATTCCAGGGTTCATCAGATCCTACGGTACTTAT
TGCCAAGAAAGCACGAGCATCAGCCGTGCCTCAATGTCGAATCTGCAAACGACGACGATCA
CGCGTCGCTCCAGTATTCCAGGGTTCATCAGATCCTACGGTACTTATTGCCAAGAAAGCACG
AGCATCAGCCGTGCCTCAATGTCGAATCTGCAAACGACGACGATCACGCGTCGCTCCAGTAT
TCCAGGGTTCATCAGATCCTACGGTACTTATTGCCAAGAAAGCACGAGCATCAGCCGTGCCT
CAATGTCGAATCTGCAAACGACGACGATCACGCGTCGCTCCAGTATTCCAGGGTTCATCA-3’
