# Supplementary material for: 3 minutes to precisely measure morphogen concentration
Source: PLoS Genet. 2018 Oct 26;14(10):e1007676. doi: 10.1371/journal.pgen.1007676 (PMC6221364; doi:10.1371/journal.pgen.1007676)
Supplement: S2 Text — (PDF) [file pgen.1007676.s002.pdf]

Determining the origin of time for each time trace

During mitosis, nuclei divide in waves, starting from the two embryo poles [2]. Therefore, nuclei at the anterior pole may produce MCP-MS2 spots earlier due to either earlier chromatin decondensation or earlier reentrance of Bcd into the nucleic space. To correct for this, we first identify the birth moment of each nucleus after mitosis. A nucleus' birth is defined as the time when the segregation from its sibling is complete (Fig. S4, A-B).

To characterize the mitotic waves, we fit the birth times t_{birth} of nuclei with siblings at position X along the AP axis in each embryo and nuclear cycle with a mirrored linear function:

$$t_{birth}([X]) = t_{meet} - \frac{|X - X_{meet}|}{k_{wave}}. \quad (1)$$

with t_{meet} and X_{meet} the moment and position two mitotic waves from the two poles meet. k_{wave} (in units of % EL/s) is the speed at which the waves propagate from the poles. An example of the fit is shown in Fig. S4C. From 8 embryos, we infer $k_{wave} \sim 2.25 \pm 1.52$ % EL/s, $X_{meet} \sim 8.5 \pm 15.7$ % EL from mid-embryo, corresponding to the wave duration of ~ 20 s.

The intensity traces in all nuclei are then trimmed so as they start at their respective nuclei birth time. The nuclei birth times are either calculated directly for nuclei with tracked siblings or inferred through the fitted t_{birth} for nuclei without tracked siblings (e.g. when nuclei move out of the imaging region).
