# Supplementary material for: 3 minutes to precisely measure morphogen concentration
Source: PLoS Genet. 2018 Oct 26;14(10):e1007676. doi: 10.1371/journal.pgen.1007676 (PMC6221364; doi:10.1371/journal.pgen.1007676)
Supplement: S3 Text — (PDF) [file pgen.1007676.s003.pdf]

Embryo alignment

Potential variations in the amount of maternal *bicoid* mRNA in the embryo's anterior pole may lead to variations in Bicoid concentrations at a given position in different embryos and thus variations in boundary position of the expression pattern. We seek to mitigate these variations by aligning the embryos' AP axis by their respective border position.

In order to do this, we first determine for each nucleus its "activity" feature (P_{active}), which takes the value of 0 if the nucleus does not produce a MS2-MCP spot during its lifetime or 1 otherwise. We then calculate the probability for a nucleus at a given position along the AP axis to have experienced transcription of the MS2 reporter (P_{ON}) as the mean of P_{active} of nuclei localized at this position The boundary position is set by inspecting where along the AP axis P_{ON} equals 0.5. The embryos' AP axes are then shifted to have their respective P_{ON} border at position 0% EL.

Note that if another feature is used as the reference for the alignment (i.e. t_{active}, μI or ΣI), the difference in embryos' relative shift does not exceed 2% EL (see Table S1) when compared to P_{active}.

All figures in the main manuscript use data from aligned embryos.
