# Supplementary material for: 3 minutes to precisely measure morphogen concentration
Source: PLoS Genet. 2018 Oct 26;14(10):e1007676. doi: 10.1371/journal.pgen.1007676 (PMC6221364; doi:10.1371/journal.pgen.1007676)
Supplement: S4 Text — (PDF) [file pgen.1007676.s004.pdf]

Quantifying the steepness of *hb* expression pattern

We quantify the steepness of the *hb* expression pattern through the features of the time traces along the AP axis: the time period during which the locus is activated (t_{active}), the integral transcription activity (ΣI) and the mean transcription rate (μI) (as defined in Fig. 6A of the manuscript) and P_{active} (defined in S3 Text).

The features are first normalized by their expected value at the anterior pole to remove variations in the embryo's growth rate and differences coming from the data acquisition process. The steepness of the feature pattern is obtained by least-square fitting the feature value along the AP axis in each embryo and nuclear cycle with a sigmoid function *f(X)*:

$$f([X]) = \frac{1}{1 + e^{H(X-X_0)/L}}. \qquad (2)$$

In Eq. 2, X is the nuclei's position. X_0 and H are the pattern's border position ($f(X)$=0.5) and the pattern steepness (i.e. the Hill coefficient) for the feature of interest. L is the decay length of the Bcd gradient, which is ~100 µm or 20 % EL [3].

The results of the fit are shown in S5 Fig (nc11), S6 Fig (nc12), S7 Fig (nc13) and in S1 Table. In nc11, due to the very short interphase duration, the pattern is barely stabilized before mitosis, leading to very large errors in the estimated H (S1 Table).
