# Supplementary material for: 3 minutes to precisely measure morphogen concentration
Source: PLoS Genet. 2018 Oct 26;14(10):e1007676. doi: 10.1371/journal.pgen.1007676 (PMC6221364; doi:10.1371/journal.pgen.1007676)
Supplement: S5 Text — (PDF) [file pgen.1007676.s005.pdf]

Evolution of the transcription pattern steepness over time

At a given time, the *hb* pattern is characterized by the snapshot proximal *hb* promoter's activity P_{bright}, which takes value of 0 if the nucleus has no active locus and 1 if the nucleus has an active locus at the given time. At a given position, P_{bright} has a mean value of P_{SPOT} (as described in the Main manuscript). We fit the values of P_{bright} along the AP axis with a sigmoid function and infer on the pattern steepness H(t) as a function of time (S8 Fig) for each nuclear cycle.
