# Supplementary material for: 3 minutes to precisely measure morphogen concentration
Source: PLoS Genet. 2018 Oct 26;14(10):e1007676. doi: 10.1371/journal.pgen.1007676 (PMC6221364; doi:10.1371/journal.pgen.1007676)
Supplement: S6 Text — (PDF) [file pgen.1007676.s006.pdf]

Models of transcription factor (TF) sensing and transcription

1) Model of TF sensing

We assume the previously suggested [4-6] model of transcription factor sensing through the multiple TF binding sites:

$$P_0 \underset{k_{-1}}{\overset{k_1[TF]}{\rightleftharpoons}} P_1 \underset{k_{-2}}{\overset{k_2[TF]}{\rightleftharpoons}} P_2 \dots \underset{k_{-N}}{\overset{k_N[TF]}{\rightleftharpoons}} P_N . \tag{3}$$

N is the number of operator sites for TF binding. P_i is the promoter state when bound by i transcription factor (TF) molecules. The operator sites are assumed to be identical, thus do not need to keep track of which operator sites are currently occupied in P_i. $[TF]$ is the TF concentration normalized by the TF concentration at the boundary position X_0. In the case of the Bcd-*hb* system, $[TF]$ follows a decaying gradient from the anterior with the decay length L ~ 100 μm or 20 % EL [3]:

$$[TF] = e^{(X-X_0)/L}. \tag{4}$$

k_i and k_{-i} are the binding and unbinding rate constants respectively. We assume that there is no cooperativity in the searching of the TF for the operator sites: when the promoter is at state P_{i-1}, TFs can bind to any of the remaining free operator sites independently at a rate:

$$k_i = (N - i + 1)/\tau_{bind}. \tag{5}$$

τ_{bind} is the time for a free operator to be bound by any TF at the *hb* expression boundary (X=X_0, $[TF]$=1). Assuming that the TF can only search for OS by diffusing in the nucleus and that each collision between TF and OS is one successful binding event, τ_{bind} depends on the diffusion coefficient of the TF (D ~ 7.7 μm^2/s [7] the absolute TF concentration at the boundary (c ~ 11.2 molecules/μm^3) [8] and the size of the operator site (a ~ 0.003 μm [6]).

$$\tau_{bind} = \frac{1}{Dac} \sim 4\ s. \tag{6}$$

We call $P(P_i,X,t)$ the probability of the promoter to be in state P_i at time t and position X. The time evolution of $P(P_i,X,t)$ is given by:

$$\begin{aligned}\frac{\partial P(P_i,X,t)}{\partial t} &= k_i e^{(X-X_0)/L} P(P_{i-1},X,t) + k_{-(i+1)} P(P_{i+1},X,t) \\ &\quad -(k_{-i} + k_{i+1} e^{(X-X_0)/L}) P(P_i,X,t)\end{aligned} . \tag{7}$$

For convenience, we define the effective equilibrium constants $\widetilde{K}_i = \sum_{j=i}^{i} k_j/k_{-j}$ for i>0 and $\widetilde{K}_i = 1$ for i=0. By solving the linear differential equations corresponding in Eq. 7, we have the probability of the promoter in state P_i at steady state at a given position X [9]:

$$P(P_i, X, t=\infty) = \frac{\overline{K_i} e^{i(X-X_0)/L}}{\sum_{j=0}^{N} \overline{K_j} e^{j(X-X_0)/L}}. \quad (8)$$

In this work, we consider the "all-or-nothing" case: the *hb* gene becomes active only when all the OS of the *hb* promoter are bound ($P_{active} \equiv P_N$).

2) Transcription initiation

While the gene is activated (i.e. the promoter is fully bound by TF), RNAP can bind stochastically to the promoter and initiate transcription at a rate $\lambda \sim 0.15\ s^{-1}$. In the initiation process, it occupies the promoter for a duration $t_{block} \sim 6\ s$ [10] preventing the binding of another RNAP to the promoter until the RNAP frees the promoter site. The values of λ and τ_{block} are extracted from the transcription dynamics of *hb* at the anterior pole [11].

3) Transcription elongation

Promoter escape is followed by a deterministic transcription elongation process [12] with a rate constant 40bp/s [13]. During this process, MCP-GFP can quickly bind to the newly transcribed MS2 binding sites on the nascent RNA.

We denote $L(t)$ the number of MS2-MCP binding sites on a nascent RNA at time t after its transcription initiation. $L(t)$ depends only on the reporter gene construct. In this work, with the ms2 binding site array located at the 3" end of the reporter gene, $L(t)$ is given in S10 Fig.

The intensity of the transcription loci is therefore given by the convolution of the transcription initiation signal $I_{RNAP}(t)$ with the reporter gene configuration function $L(t)$:

$$I(t) = I_0 I_{RNAP}(t) * L(t). \quad (9)$$

Here, I_0 is the intensity of a single bound MS2-MCP binding site.

4) The pattern steepness and the promoter dynamics out of steady state

For a given set of kinetic parameters $\overline{k} = [k_i, k_{-i}]$ in Eq. 3, we quantify the pattern steepness and the evolution of the promoter mean activity over time. We only focus on the boundary position (X=X_0) where the gene is 50% activated at steady-state ($P(P_{active}, X_0, \infty)$=0.5).

Pattern steepness

We calculate the pattern steepness H from the slope of the promoter activity pattern at the boundary position (X=X_0) at steady state:

$$H = \frac{4}{L}\left(\frac{\partial P(P_N, X, \infty)}{\partial X}\right)_{X=X_0}. \quad (10)$$

Promoter dynamics of steady state

The vector $s(t)=[P(P_0,X,t), P(P_0,X,t),\ldots P(P_0,X,t)]^T$ describes the probability for nuclei at a position X and time to be in a given promoter state. The change in the vector $s(t)$ is described by the transition matrix U, whose elements are defined by the stochastic equations in Eq. 7 evaluated at the boundary position ($X=X_0$). At time $t=0$, we assume that the promoter is free of TF: $s(0)=[1,0\ldots,0]^T$ and the TF interact with OS of the promoter. The mean promoter activity at time t is given by:

$$s(t) = u(0)e^{Ut} \quad (11)$$

We define a vector α indicating which promoter state is associated with the target being activated. In the case of "all-or-nothing" ($P_{active} \equiv P_N$), $\alpha=[0,0\ldots,1]^T$. The distribution of nuclei with active loci is given by:

$$s_{active}(t) = \alpha^T.s(0)e^{Ut}. \quad (12)$$

5) Fitting the transcription pattern

We find the kinetic parameter set $\overline{k^*} = [k_i^*, k_{-i}^*]$ that matches the observed Hill coefficient and the promoter state probability at the boundary ($X=X_0$) at near steady state (330 s from the beginning of nuclear cycle or ~180 s after first spot appearances). For simplicity, we approximate the probability of the activated gene s_{active}(t=180 s) by the probability of the spot appearance in each nuclei P_{SPOT} at 330 s following mitosis.

From the measurements, we infer the Hill coefficient H ~ 7.1 ± 0.53, s_{active}(180 s) ~ 0.45 ± 0.02.

We fit the model's Hill coefficient and the promoter active state distribution to the data using least square fit, weighted by the margin of error. The value of $\overline{k^*}$ can be found by minimizing the objective:

$$\overline{k^*} = \arg\min_{\overline{k}} \left[\left(\frac{\overline{H}(k)-7.1}{0.53}\right)^2 + \left(\frac{\overline{s}(k)-0.45}{0.02}\right)^2\right]. \quad (13)$$

Where $\overline{H}(\overline{k})$ and $\overline{s}(\overline{k})$ are respectively the steepness and the probability of activated gene calculated numerically from Eq. 10 and 12 for any given parameter set $\overline{k}$.

Due to the model complexity and the high number of parameters involved with $N \geq 6$, $\overline{k^*}$ is obtained by a brute force search, followed by local optimization (~ 10^5 iterations for each value of N). The unbinding rates k_{-i} are randomized from 10^{-20} s^{-1} to 10^{20} s^{-1} while the binding rates k_i are kept constant (Eq. 5).

The fitted kinetic parameters are shown in S2 Table.

Given the p-value of the likelihood ratio test between the models and the Bayesian Information Criterion (BIC) for each model, we find that increasing N up to 9 results in significant better fit of the model with data. Increasing N beyond 9 does not improve the fit significantly.

6) Stochastic simulations

We use the Stochastic Simulation Algorithm [14, 15] to simulate the promoter dynamics under the regulation of the TF and the timing of the transcription initiation events by RNAP $I_{RNAP}(t)$. The trajectory of $I_{RNAP}(t)$ is then convoluted with the gene configuration function $L(t)$ to achieve the spot intensity $I(t)$ over time. An example of the intensity trace is shown in S11 Fig.

At each position along the AP axis, with the fitted kinetic parameters, we simulate 500 nuclei intensity traces, from which the heatmap of P_{SPOT} over time and along AP axis can be constructed (Fig. 8 in main Manuscript).
