# Supplementary material for: 3 minutes to precisely measure morphogen concentration
Source: PLoS Genet. 2018 Oct 26;14(10):e1007676. doi: 10.1371/journal.pgen.1007676 (PMC6221364; doi:10.1371/journal.pgen.1007676)
Supplement: S7 Text — (PDF) [file pgen.1007676.s007.pdf]

Comparing the noise in positional readout between models

With the fitted parameters (S2 Table), we compare the precision of gene expression readout f_T between the case N=6, N=9 at the boundary position (X=X_0). Here, the readout is defined as the mean duration the *hb* gene is activated at steady-state:

$$f_T = \int_{t=0}^{T} \frac{1}{T} n(t). \tag{14}$$

with *n(t)* being the trajectory of the gene activity state over time. *n(t)* is 1 when the gene is activated and 0 otherwise. The relative noise in the readout CV_P is defined as follow:

$$CV_P = \frac{\delta f T}{\langle f_T \rangle} = \sqrt{\frac{\langle f_T^2 \rangle - \langle f_T \rangle^2}{\langle f_T \rangle^2}}, \tag{15}$$

in which $<f_T>$=0.5 and $<f_T^2>$ are respectively the first and second moments of the readout at the pattern's boundary (X=$X0$). Let us define a vector s_{fire} where $s_{fire,i}$=α_i.s_i. $<f_T^2>$ is calculated numerically from the transition matrix U [9]:

$$\langle f_T \rangle = \frac{2\alpha^{\mathrm{T}}}{T} \left[\int_0^T dt (T-t) e^{Ut} \right] s_{fire}, \tag{15}$$

The precision of gene expression readout between the model with N=6 and N=9 are shown in S12 Fig. Also shown is precision from the "no cooperativity" case, where interactions of TF with the binding sites are independent (k_{-i} = i.k_{-N}/N).
