# Supplementary material for: 3 minutes to precisely measure morphogen concentration
Source: PLoS Genet. 2018 Oct 26;14(10):e1007676. doi: 10.1371/journal.pgen.1007676 (PMC6221364; doi:10.1371/journal.pgen.1007676)
Supplement: S8 Text — (PDF) [file pgen.1007676.s008.pdf]

Supporting References

1. Venken K.J.T., He Y., Hoskins R.A., and Bellen H.J. (2006). P[acman]: A BAC Transgenic Platform for Targeted Insertion of Large DNA Fragments in D. melanogaster. Science *314*, 1747-1751.
2. Deneke V.E., Melbinger A., Vergassola M., and Di Talia S. (2016). Waves of Cdk1 Activity in S Phase Synchronize the Cell Cycle in Drosophila Embryos. Developmental cell *38*, 399-412.
3. Gregor T., Wieschaus E.F., McGregor A.P., Bialek W., and Tank D.W. (2007). Stability and Nuclear Dynamics of the Bicoid Morphogen Gradient. Cell *130*, 141-152.
4. Driever W., and Nusslein-Volhard C. (1989). The bicoid protein is a positive regulator of hunchback transcription in the early Drosophila embryo. Nature *337*, 138-143.
5. Estrada J., Wong F., DePace A., and Gunawardena J. (2016). Information Integration and Energy Expenditure in Gene Regulation. Cell *166*, 234-244.
6. Gregor T., Tank D.W., Wieschaus E.F., and Bialek W. (2007). Probing the Limits to Positional Information. Cell *130*, 153-164.
7. Porcher A., Abu-Arish A., Huart S., Roelens B., Fradin C., and Dostatni N. (2010). The time to measure positional information : maternal Hunchback is required for the synchrony of the Bicoid transcriptional response at the onset of zygotic transcription. Development *137*, 2795-2804.
8. Abu-Arish A., Porcher A., Czerwonka A., Dostatni N., and Fradin C. (2010). Fast mobility of Bicoid captured by flourescent correlation spectroscopy : implication for the rapid establishment of its gradient Biophysical Journal *99*, L33-L35.
9. Tran H., Desponds J., Perez-Romero C.A., Coppey M., Fradin C., Dostatni N., and Walczak A.M. (in prep). Precision in a rush: hunchback pattern formation in a limited time. To be submitted and deposited on the Bioarxiv shortly.
10. Garcia Hernan G., Tikhonov M., Lin A., and Gregor T. (2013). Quantitative Imaging of Transcription in Living Drosophila Embryos Links Polymerase Activity to Patterning. Current Biology *23*, 2140-2145.
11. Desponds J., Tran H., Ferraro T., Lucas T., Perez Romero C., Guillou A., Fradin C., Coppey M., Dostatni N., and Walczak A.M. (2016). Precision of Readout at the hunchback Gene: Analyzing Short Transcription Time Traces in Living Fly Embryos. PLOS Computational Biology *12*, e1005256.
12. Coulon A., Ferguson M.L., de Turris V., Palangat M., Chow C.C., and Larson D.R. (2014). Kinetic competition during the transcription cycle results in stochastic RNA processing. eLife *3*, e03939.
13. Fukaya T., Lim B., and Levine M. (2017). Rapid Rates of Pol II Elongation in the Drosophila Embryo. Current biology : CB *27*, 1387-1391.
14. Gillespie D.T. (1976). A general method for numerically simulating the stochastic time evolution of coupled chemical reactions. Journal of Computational Physics *22*, 403-434.
15. Lloyd-Price J., Gupta A., and Ribeiro A.S. (2012). SGNS2: a compartmentalized stochastic chemical kinetics simulator for dynamic cell populations. Bioinformatics (Oxford, England) *28*, 3004-3005.
