# Supplementary figures and images for: 3 minutes to precisely measure morphogen concentration
Source: PLoS Genet. 2018 Oct 26;14(10):e1007676. doi: 10.1371/journal.pgen.1007676 (PMC6221364; doi:10.1371/journal.pgen.1007676)

**S1 Fig.**

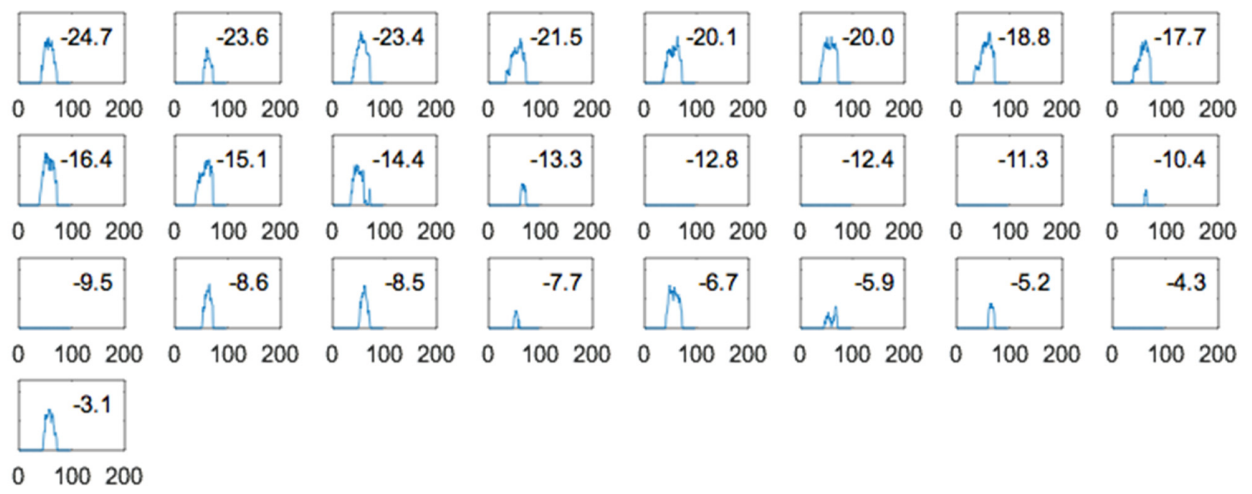

Supplement: S1 Fig — The time traces are sorted by their respective nuclei position, which is shown in the boxes in % EL (Position 0 corresponding to the middle of the embryo). Nuclei with position beyond -3.1% EL (last shown nucleus) have no spots. Horizontal axis: time in seconds. Vertical axis: spot fluorescent intensity in arbitrary units. (PDF) [file pgen.1007676.s009.pdf]

S2 Fig

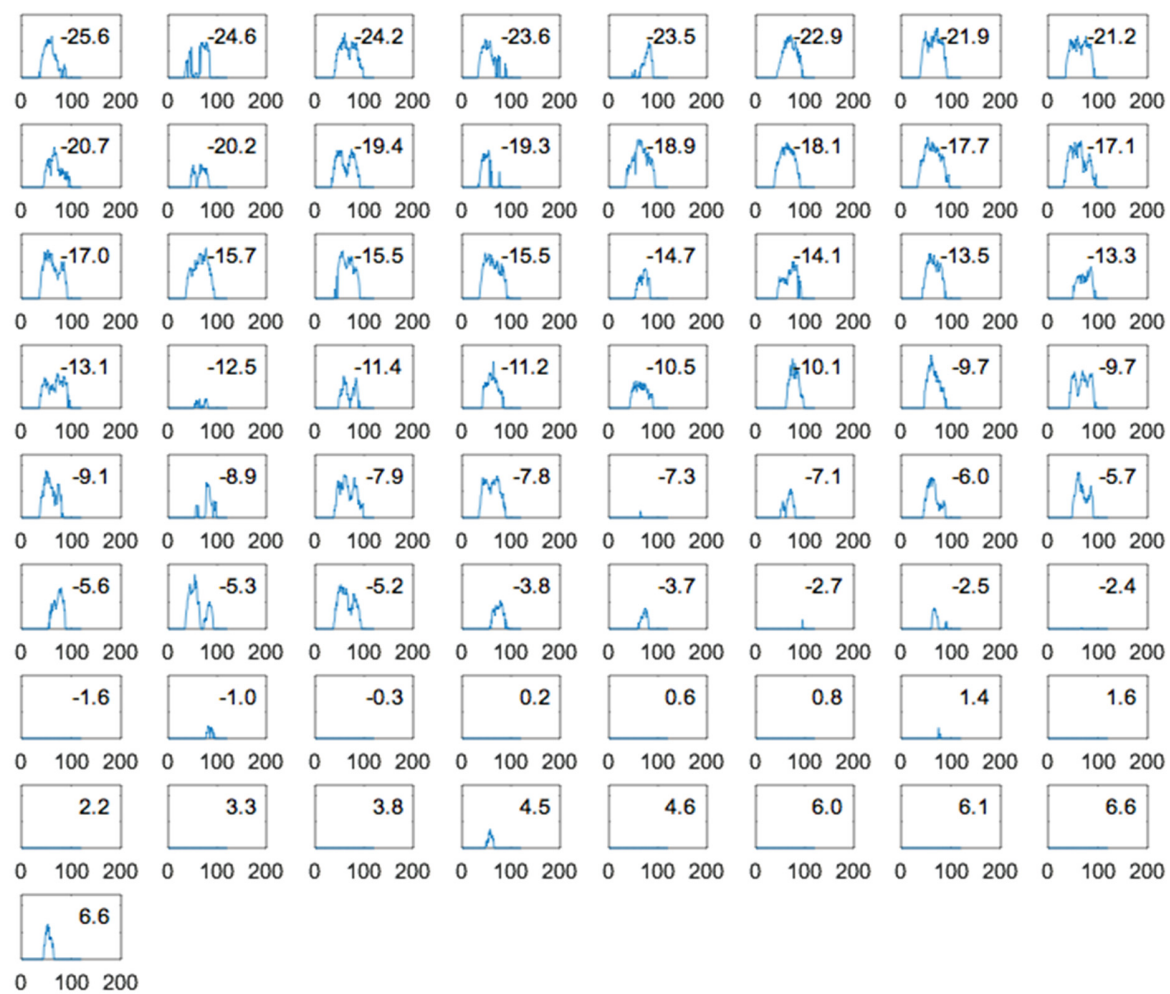

Supplement: S2 Fig — The time traces are sorted by their respective nuclei position, which is shown in the boxes in % EL (Position 0 corresponding to the middle of the embryo). Nuclei with position beyond 6.6% EL (last shown nucleus) have no spots. Horizontal axis: time in seconds. Vertical axis: spot fluorescent intensity in arbitrary units. (PDF) [file pgen.1007676.s010.pdf]

**S3 Fig.**

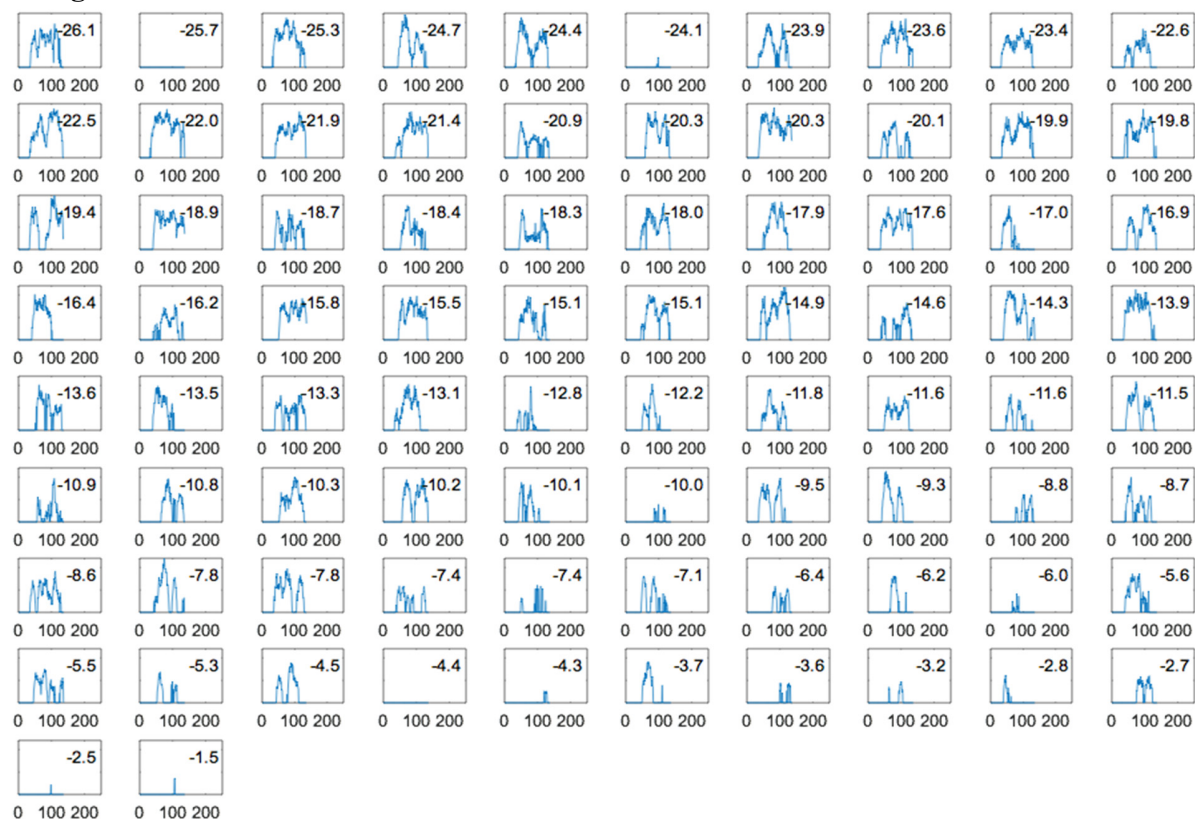

Supplement: S3 Fig — The time traces are sorted by their respective nuclei position, which is shown in the boxes in % EL (Position 0 corresponding to the middle of the embryo). Nuclei with position beyond -1.5% EL (last shown nucleus) have no spots. Horizontal axis: time in seconds. Vertical axis: spot fluorescent intensity in arbitrary units. (PDF) [file pgen.1007676.s011.pdf]

S4 Fig.

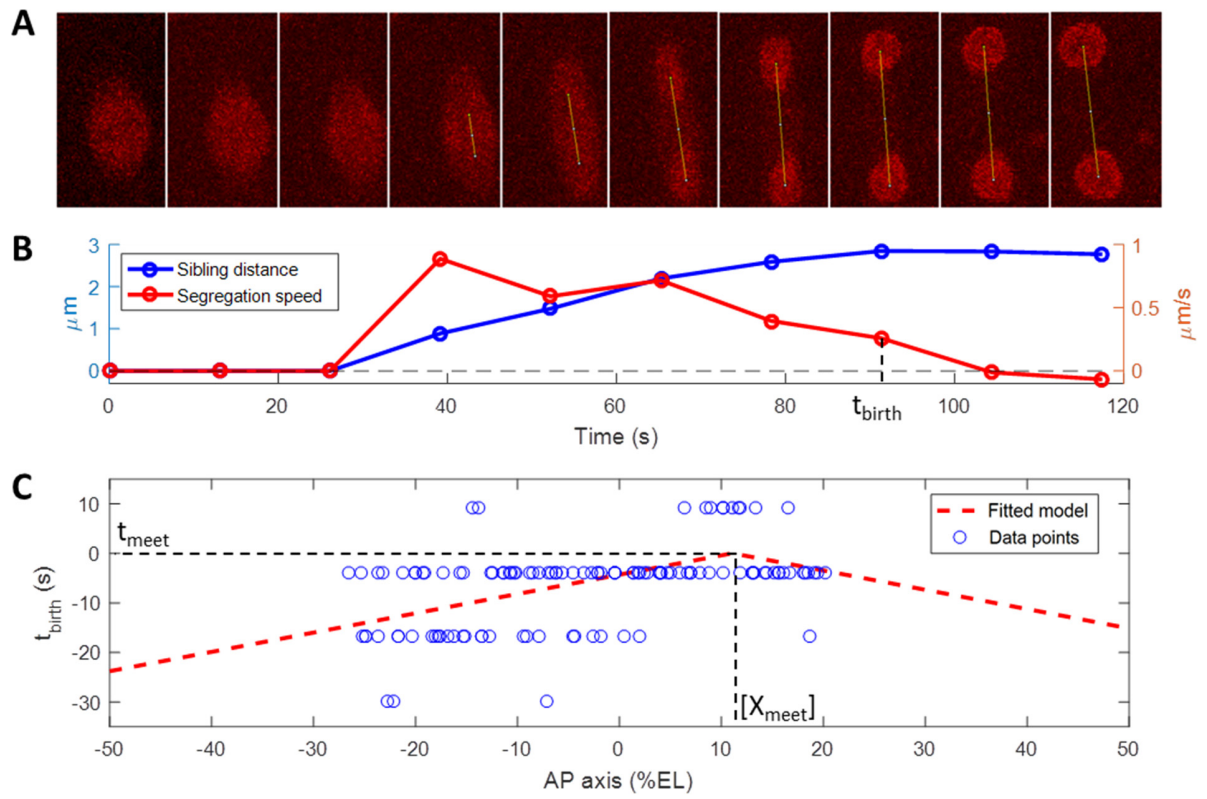

Supplement: S4 Fig — (A) Examples of frame-by-frame monitoring of two sibling nuclei after mitosis. The time interval between frames is 13.05 s. The yellow line is drawn automatically to connect the two siblings’ centroids once division is detected. (B) The distance between the centroids (blue line) and its derivative (red line) over time. The nuclei’s birth is set as the time when the speed of segregation between the sibling nuclei decreases to near-zero. (C) Examples of the nuclei birth time tbirth along AP axis. Shown is the tbirth extracted from the movies (blue circles) and from the fitted model in Eq 1 (red dashed line). The y axis (tbirth) is shifted so as the two mitotic waves from the two poles meet at tmeet = 0. (PDF) [file pgen.1007676.s012.pdf]

S5 Fig.

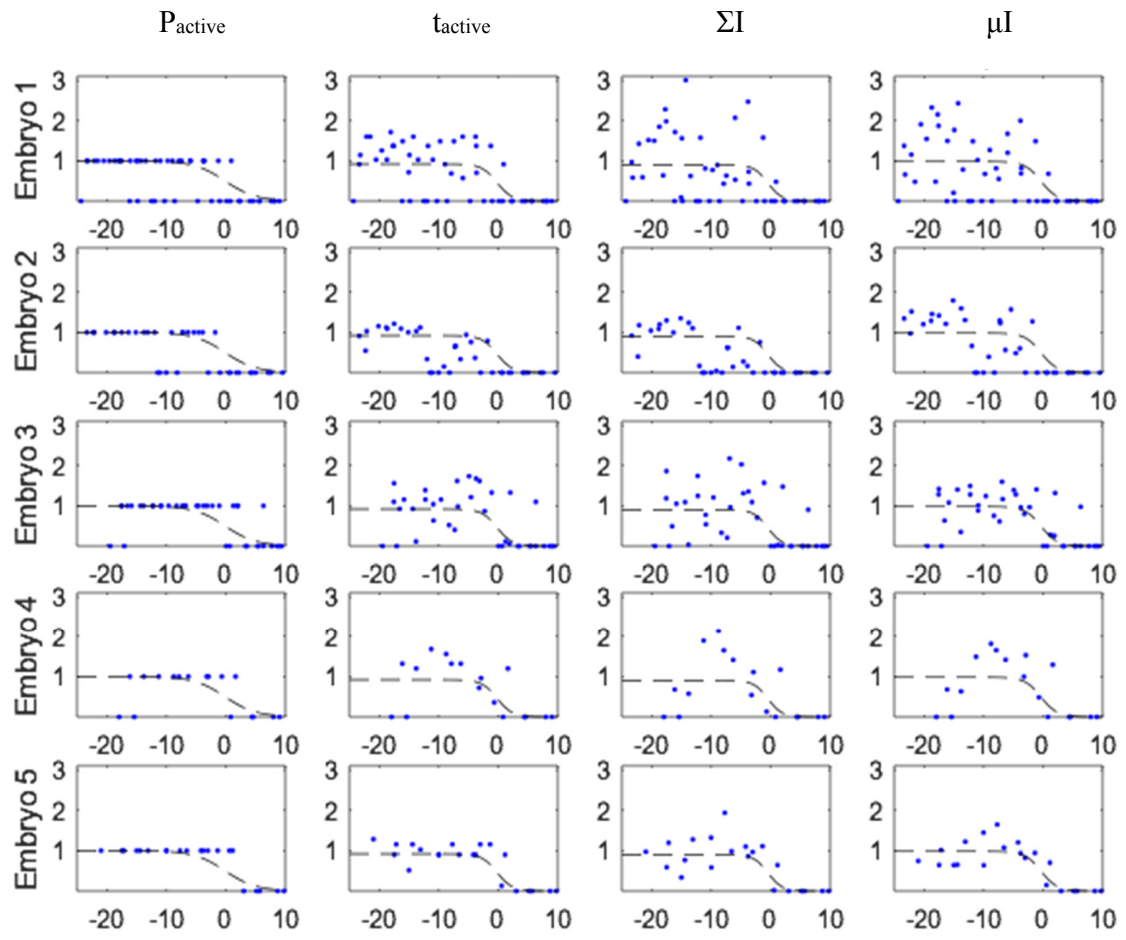

Supplement: S5 Fig — The fitted curves (dashed black lines) are shown with data points (blue dots). Each data point corresponds to a single trace feature value. The horizontal axis is the AP axis in % EL. (PDF) [file pgen.1007676.s013.pdf]

S6 Fig.

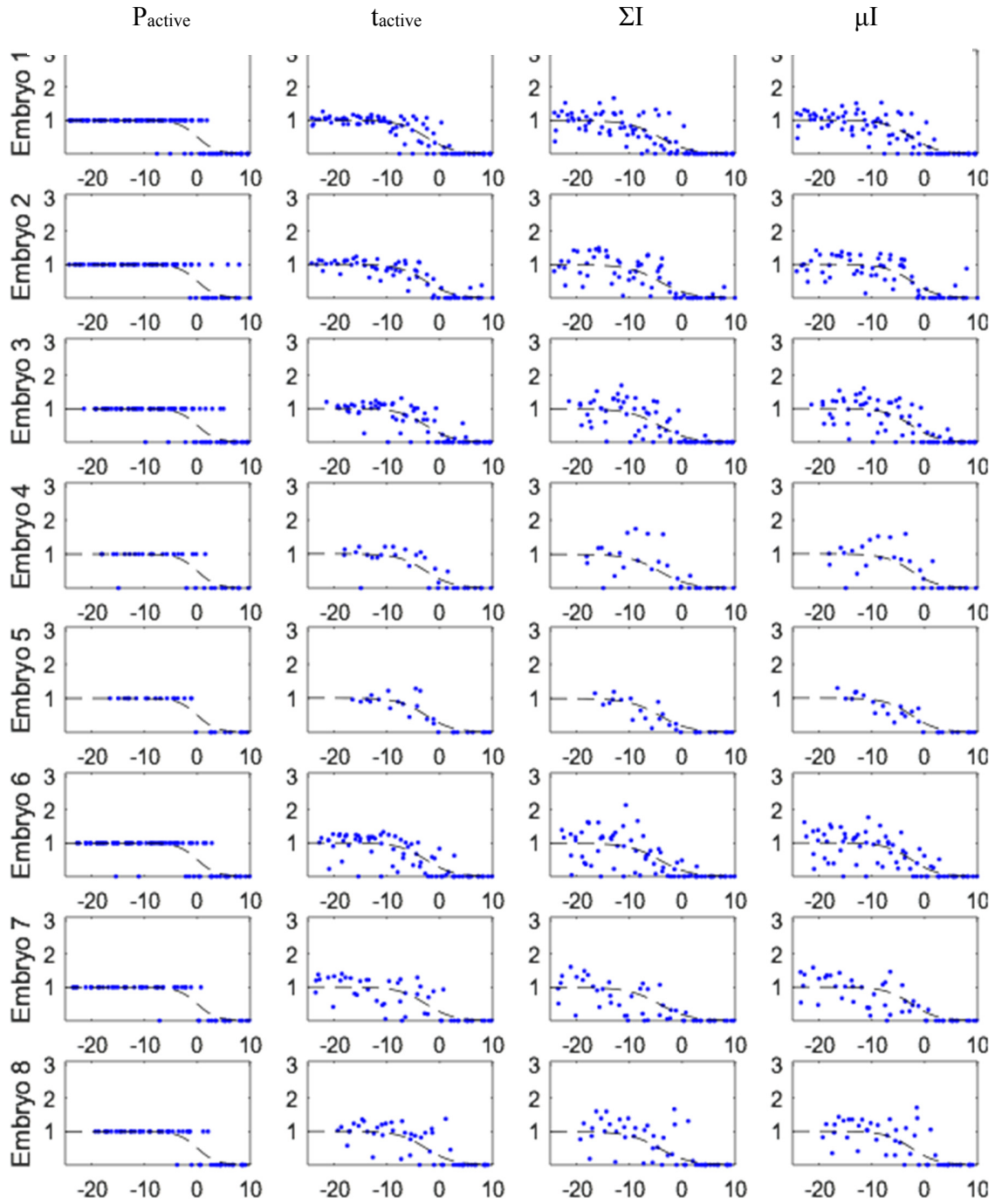

Supplement: S6 Fig — The fitted curves (dashed black lines) are shown with data points (blue dots). Each data point corresponds to a single trace feature value. The horizontal axis is the AP axis in % EL. (PDF) [file pgen.1007676.s014.pdf]

S7 Fig.

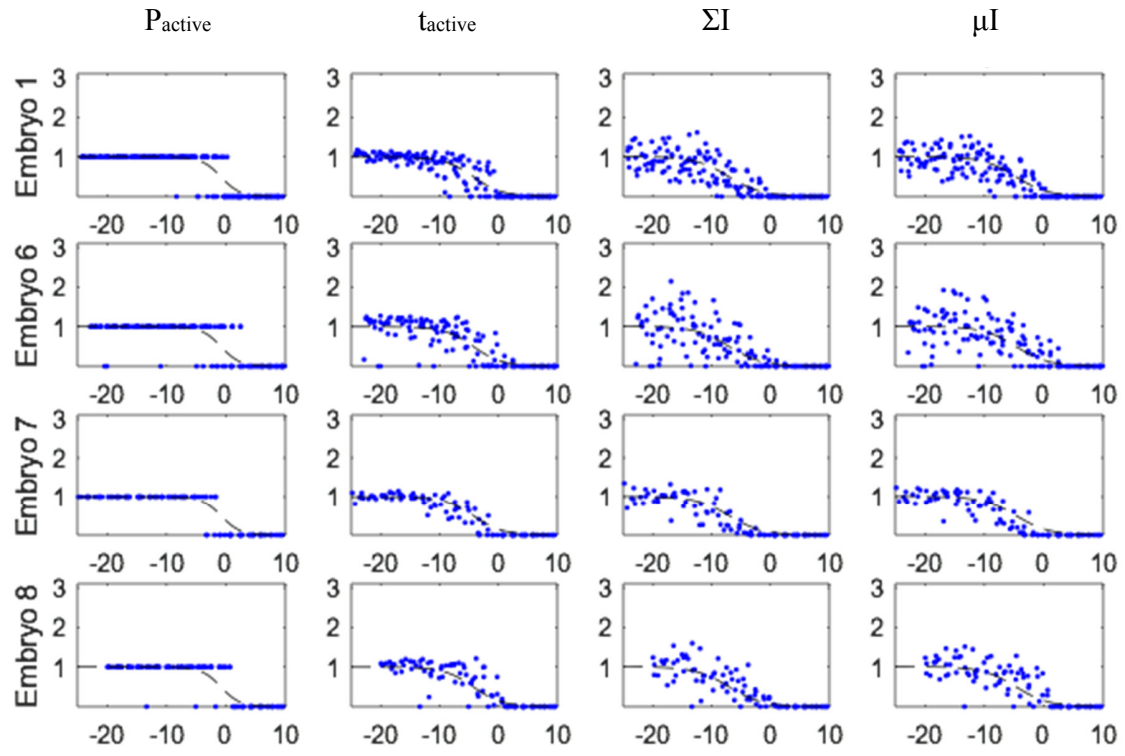

Supplement: S7 Fig — The fitted curves (dashed black lines) are shown with data points (blue dots). Each data point corresponds to a single trace’s feature value. The horizontal axis is the AP axis in % EL. (PDF) [file pgen.1007676.s015.pdf]

**S8 Fig.**

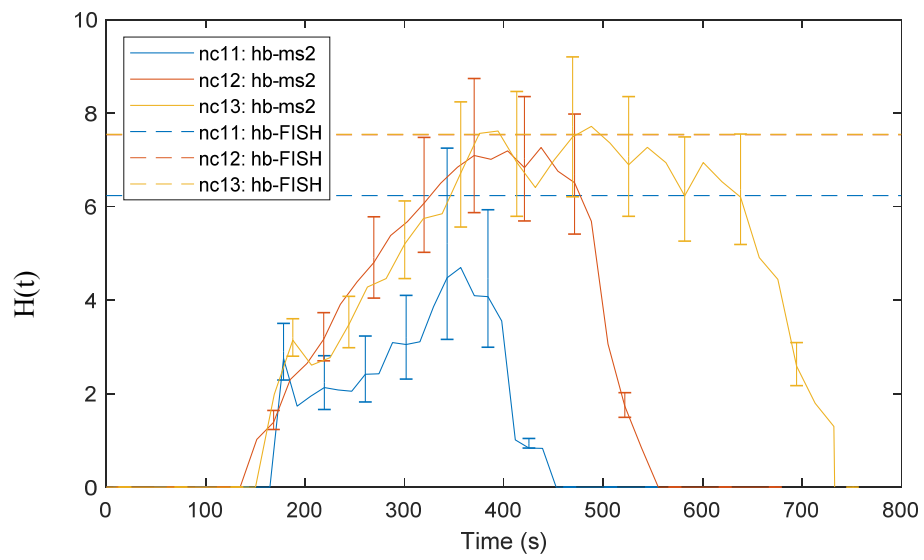

Supplement: S8 Fig — Shown for nc 11 (blue solid line), nc 12 (red solid line) and nc 13 (yellow solid line) along with the margins of errors (p-value = 0.05). Also shown (dashed lines) are the Hill coefficients extracted from FISH data in for the respective cycles. The coefficients from FISH in nc12 and nc13 are almost identical. (PDF) [file pgen.1007676.s016.pdf]

S9 Fig.

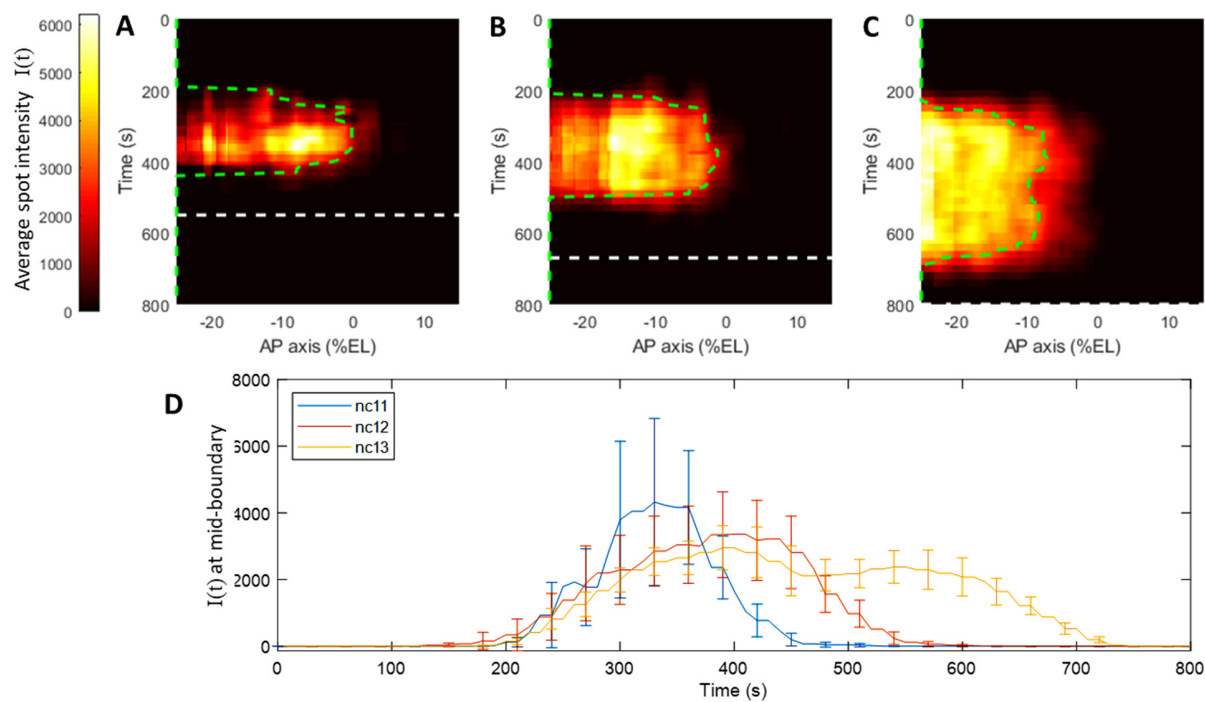

Supplement: S9 Fig — A-C: The average spot intensity I(t) is indicated by a heat map (color scale on the right) horizontally as a function of position along the AP axis (0% EL positioned PON boundary at nc12) and vertically as a function of time (s) fixing the origin at the onset of interphase for each nucleus (see details in S2 text and S4 Fig). For each cycle (A: nc11; B: nc12; C: nc13), the end of interphase (onset of next mitosis) is indicated by a dashed line (white). The green dashed line indicates the position of the expression boundary (I(t) equals half the average spot intensity at the anterior pole) over time. D: I(t) as a function of time (s) at mid-boundary position. The first hints of transcription are observed at mid-boundary position ~ 170 s after the onset of interphase (lower limit of the light blue zone) and steady state is reached at ~ 350 s. Boundary formation reaches steady state in ~ 180 s. Data were obtained from 5 (nc11), 8 (nc12) and 4 (nc13) embryos. Embryos were aligned spatially fixing the origin of the axis at boundary position (PON) at nc12 and the origin of time was calculated for each nuclei as the origin of its respective cycle (see S2 Text and S4 Fig). (PDF) [file pgen.1007676.s017.pdf]

**S10 Fig.**

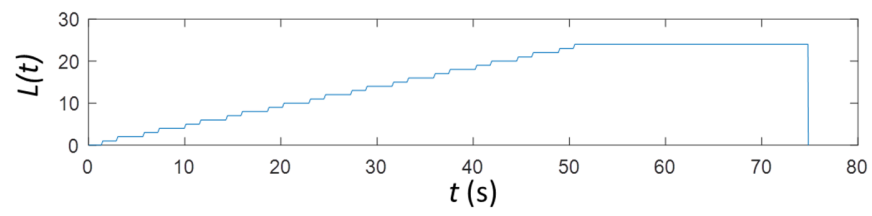

Supplement: S10 Fig — L(t) is the number of MS2-MCP binding sites on a nascent RNA at time t after its transcription initiation. (PDF) [file pgen.1007676.s018.pdf]

**S11 Fig.**

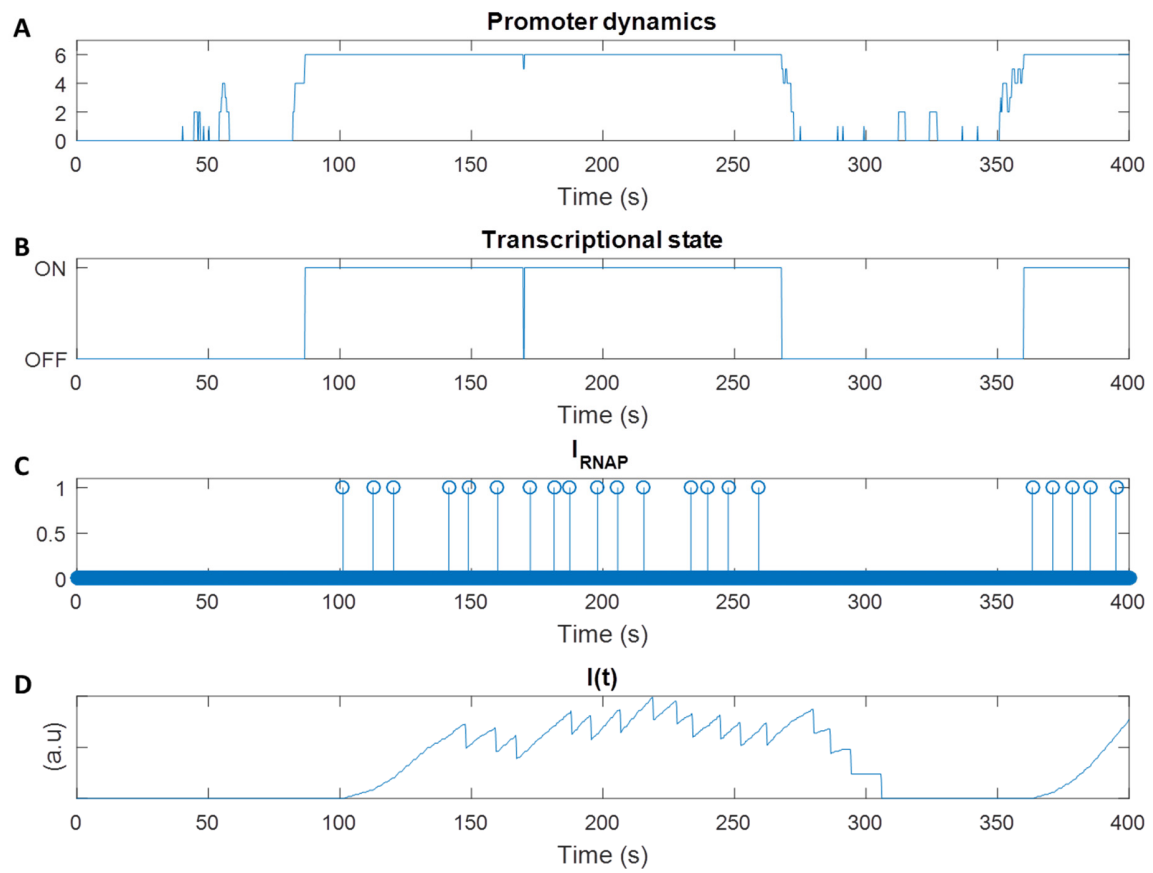

Supplement: S11 Fig — (A) The number of bound TF molecules to the promoter over time. (B) The gene transcriptional state given the number of bound molecules to the promoter. The gene is turned ON when the promoter is fully bound by TF. (C) Occurrences of transcription initiation events IRNAP(t), corresponding to (B). (D) Transcription loci intensity I(t), corresponding to (C). (PDF) [file pgen.1007676.s019.pdf]

**S12 Fig.**

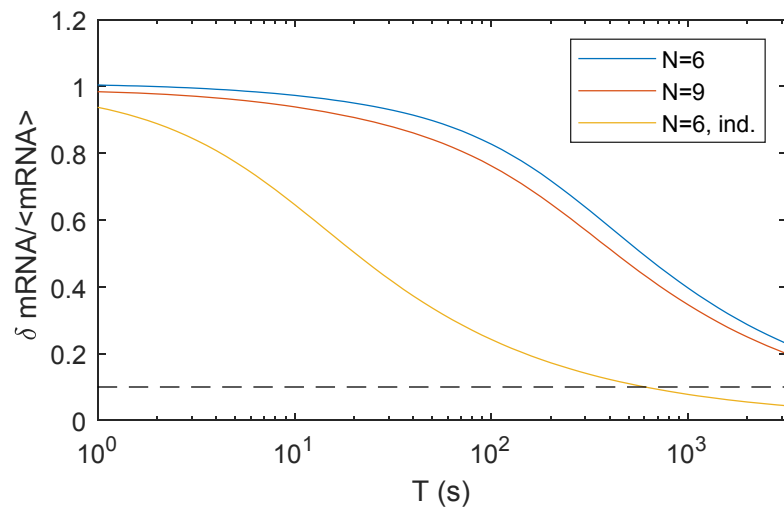

Supplement: S12 Fig — For fitted model with N = 6 (blue line), fitted model with N = 9 (red line) and “no cooperativity” model (yellow line). (PDF) [file pgen.1007676.s020.pdf]
