# Supplementary material for: 3 minutes to precisely measure morphogen concentration
Source: PLoS Genet. 2018 Oct 26;14(10):e1007676. doi: 10.1371/journal.pgen.1007676 (PMC6221364; doi:10.1371/journal.pgen.1007676)
Supplement: S1 Table — The position of the feature pattern border (X0, in units of % EL), the pattern steepness (H) and their respective confidence interval (in brackets) for the locus activity (Pactive), the time period during which the locus is activated (tactive), the integral transcription activity (ΣI) and the mean transcription rate (μI). The data are inferred from all aligned embryos in the respective cycles. (PDF) [file pgen.1007676.s021.pdf]

S1 Table

Nuclear cycle	Feature	P_{active}	t_{active}	ΣI	μI
11	X_0 (% EL)	-0.1 (-1.2;1)	-0.2 (-1.2;0.8)	-0.5 (-1.8;0.8)	-0.3 (-1.4;0.8)
	H	7.7 (4.2;14.0)	16.0 (8.1;∞)	17.2 (7.2; ∞)	15.0 (7.8; ∞)
12	X_0 (% EL)	0 (-0.3;0.3)	-2.7 (-3.1;-2.3)	-4.3 (-4.9;-3.7)	-2.5 (-3.0;-2.0)
	H	11.2 (9.2;13.9)	7.8 (6.6;9.3)	6.9 (5.8;8.5)	8.1 (6.7;10.1)
13	X_0 (% EL)	-0.9 (-1.2;-0.6)	-4.3 (-4.6;4.0)	-7.0 (-7.5;-6.5)	-4.7 (-5.2;-4.3)
	H	12.6 (10.7 15.0)	8.2 (7.2;9.3)	7.1 (6.2;8.3)	7.3 (6.3;8.4)
