# Supplementary material for: 3 minutes to precisely measure morphogen concentration
Source: PLoS Genet. 2018 Oct 26;14(10):e1007676. doi: 10.1371/journal.pgen.1007676 (PMC6221364; doi:10.1371/journal.pgen.1007676)
Supplement: S2 Table — Shown is the p-value of the likelihood ratio test between the fitted model of N and N-1 OS. Also shown is the Bayesian Information Criterion (BIC) for each model. The data for the fitting is pulled from all embryos in all nuclear cycles. (PDF) [file pgen.1007676.s022.pdf]

S2 Table

$\overline{k^*}$	*N*=6	*N*=7	*N*=8	*N*=9	*N*=10	*N=6 Ind.*
k_{-1} (s^{-1})	21.34	31.02	1.12	4.20	0.03	0.48
k_{-2} (s^{-1})	1.50	0.92	16.15	8.14	36.46	0.97
k_{-3} (s^{-1})	0.51	0.51	1.87	2.01	0.39	1.46
k_{-4} (s^{-1})	2.47	1.60	2.12	1.59	4.33	1.94
k_{-5} (s^{-1})	0.19	0.82	1.25	1.20	1.59	2.43
k_{-6} (s^{-1})	0.016	0.84	0.31	0.47	3.10	2.91
k_{-7} (s^{-1})	/	0.011	0.80	1.85	0.40	/
k_{-8} (s^{-1})	/	/	0.013	0.19	0.76	/
k_{-9} (s^{-1})	/	/	/	0.025	0.31	/
k_{-10} (s^{-1})	/	/	/	/	0.023	/
$H(\bar{k})$	5.15	5.48	6.11	6.56	6.69	1.31
$s(\bar{k})$	0.43	0.45	0.43	0.44	0.44	0.48
p-value	1	0.0050	0.0044	0.0133	0.645	/
BIC	45.7	40.7	35.4	32.1	24.7	212.6
